# Novel Anatomical Guidelines on Botulinum Neurotoxin Injection for Wrinkles in the Nose Region

**DOI:** 10.3390/toxins14050342

**Published:** 2022-05-15

**Authors:** Kyu-Ho Yi, Ji-Hyun Lee, Hye-Won Hu, Hee-Jin Kim

**Affiliations:** 1 COVID-19 Division, Wonju Public Health Center, Wonju-si 26417, Korea; kyuho90@daum.net; 2 Division in Anatomy and Developmental Biology, Department of Oral Biology, Human Identification Research Institute, BK21 PLUS Project, Yonsei University College of Dentistry, 50-1 Yonsei-ro, Seoul 03722, Korea; jh_anatomy@yuhs.ac (J.-H.L.); wonhuh@yuhs.ac (H.-W.H.)

**Keywords:** nasalis muscle, procerus muscle, levator labii superioris alaeque muscles, botulinum neurotoxin, bunny line, horizontal radix line, facial wrinkle, injection point

## Abstract

Botulinum neurotoxin injection surrounding the nose area is frequently used in aesthetic settings. However, there is a shortage of thorough anatomical understanding that makes it difficult to treat wrinkles in the nose area. In this study, the anatomical aspects concerning the injection of botulinum neurotoxin into the nasalis, procerus, and levator labii superioris alaeque muscles are assessed. In addition, the present knowledge on localizing the botulinum neurotoxin injection point from a newer anatomy study is assessed. It was observed that, for the line-associated muscles in the nose region, the injection point may be more precisely defined. The optimal injection sites are the nasalis, procerus, and levator labii superioris alaeque muscles, and the injection technique is advised. We advise the best possible injection sites in association with anatomical standards for commonly injected muscles to increase efficiency in the nose region by removing the wrinkles. Similarly, these suggestions support a more precise procedure.

## 1. Introduction

**Figure 1:**
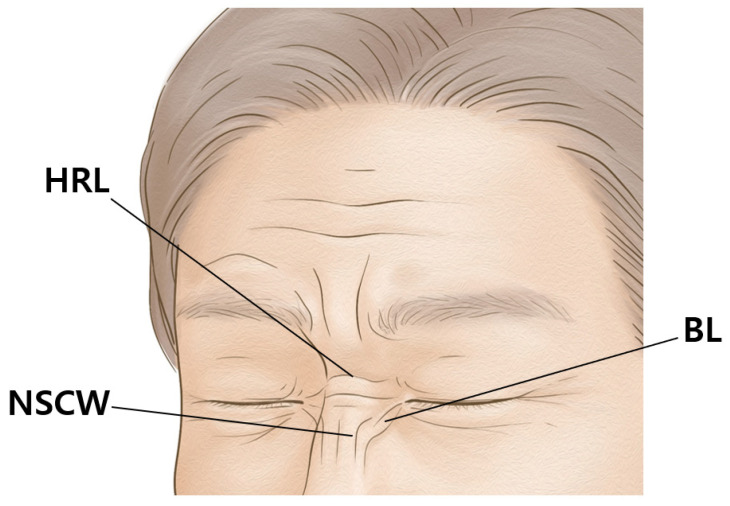
The wrinkles of the nose region are the bunny lines (BL), horizontal radix line (HRL), and nasal side wall scrunch wrinkles (NSCW).

Botulinum neurotoxin (BoNT) prevents neural connections by stimulating the release of acetylcholine at the motor endplates, obstructing the muscle from contracting [1,2]. In aesthetic clinics, BoNT is commonly used primarily to eliminate wrinkles in the nose region by weakening the muscles involved in facial expression, such as the procerus, nasalis, and levator labii superioris alaeque nasi muscles. The primary aesthetic concerns in the nose region are the bunny lines, horizontal radix line, and nasal side wall scrunch wrinkles for many individuals (Figure 1).

As wrinkle removal using BoNT is being performed more often, the adverse effects, such as paralysis of the nearby muscles, diplopia, ptosis, and samurai eyebrows have been reported [3]. When treating wrinkles with BoNT in the nasal region, significant problems, such as diplopia may result from unintended paralysis of the rectus inferior or medialis [4,5]. To prevent these side effects, the injection should be administered at an anatomically accurate location of the targeting muscle, and the initial treatment should be at a reduced dosage. 

Another factor that should be considered is that large doses and repetitive injections of BoNT create antibodies, leading to inadequate treatment outcomes [6,7,8,9]. According to previous research, antibody formation differs with types of botulinum neurotoxin [10,11].

**Figure 2:**
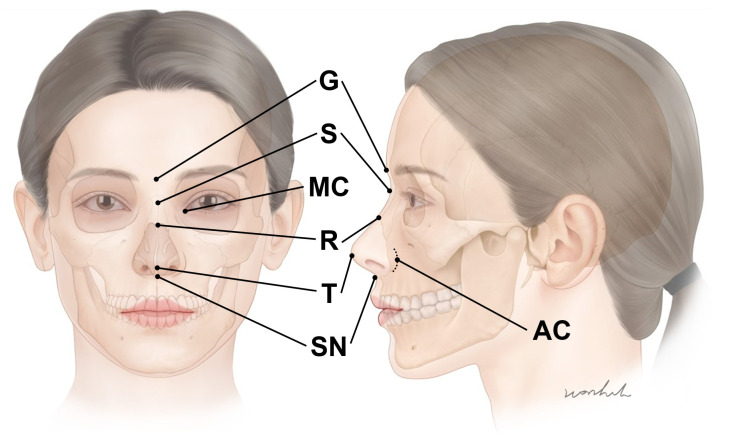
The external anatomical landmarks of the nose regions. G—glabella; S—sellion; R—rhinion; T—nose tip; SN—subnasale; MC—medial canthus; AC—alar crease.

Numerous studies on BoNT injection points in muscles have previously been published on external anatomical standards (Figure 2) [12,13,14,15,16,17,18,19,20,21,22,23,24]. We searched for articles using the following keywords: “botulinum neurotoxin in nose region” and “side effect of botulinum neurotoxin injection in nose wrinkle” on Pubmed and Scopus. A total of 16 articles and two textbooks were found; 10 articles were excluded owing to the irrelevance to this studies. The objective of this study is to propose a safe and efficient BoNT injection point and suggest injective techniques for wrinkles in the nasal region. 

## 2. The Anatomy of the Muscles in the Nasal Region

**Figure 3:**
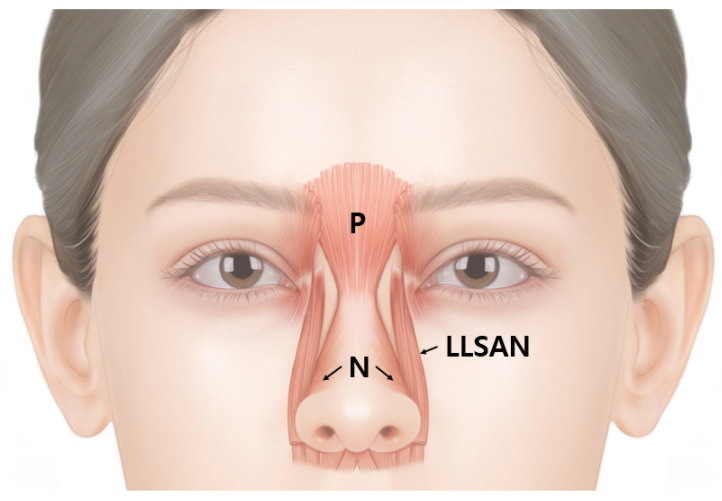
Schematic image of the procerus (P), nasalis (N), and levator labii superioris alaeque nasi (LLSAN).

**Figure 4:**
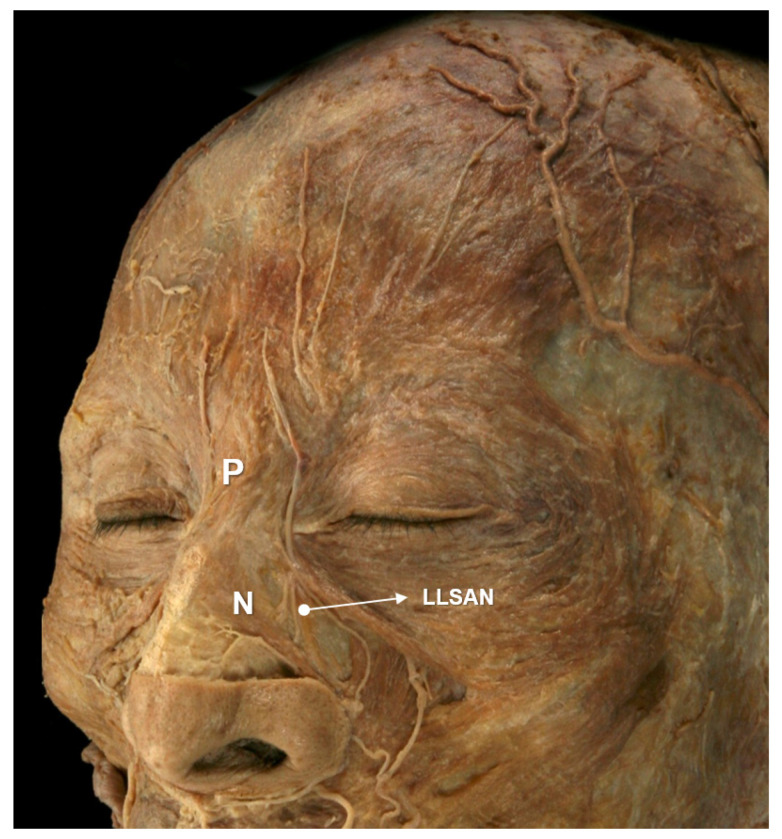
The dissected image of the procerus (P), nasalis (N), and levator labii superioris alaeque nasi (LLSAN).

The schematic and dissected images of the muscles in the nasal region are presented below (Figure 3 and Figure 4).

### 2.1. Nasalis Muscle

The nasalis muscle is composed of transverse and alar parts [25]. The transverse part of the nasalis muscle is a morphologically triangular structure originating from the maxillary canine fossa which inserts into the lateral cartilage of the nose [26]. The alar part of the nasalis is a square-like muscle that originates from the maxillary lateral incisor and inserts into the lower alar cartilage [26]. These two parts of the nasalis muscle both contribute to the narrowing of the nostrils. However, the transverse muscle contracts the nasal aperture while the alar muscle widens the nostrils.

### 2.2. Procerus Muscle

The procerus muscle originates deep from the lateral cartilage of the nose and nasal bone, inserting superficially into the skin at the glabella and radix [27]. In the glabella, the muscle fibers of the procerus muscle combine with the frontalis muscle [28]. The procerus muscle acts by pulling down the medial portion of the eyebrow while creating a transverse wrinkle between the glabella and the sellion.

### 2.3. Levator Labii Superioris Alaeque Nasi Muscle

The levator labii superioris alaeque nasi muscle is a long running muscle originating in the maxillary frontal process, and involves the nasal ala and upper lip [29]. The levator labii superioris alaeque nasi muscle can be divided into deep and superficial bellies [30]. The deep belly runs deep to the levator labii superioris muscle, whereas the superficial belly runs superficial to the levator labii superioris muscle [29]. 

## 3. Injection Techniques

### 3.1. Horizontal Radix Line

The horizontal radix lines are mainly caused by the procerus muscle; thereby, targeting the procerus muscle is the critical injection point. A dose of 2 U should be injected into the nasal dorsum. An accurate point should be located in the middle of the glabella and sellion. The glabella is the midline bony prominence between the frontal bone and supraciliary arches. In addition, the glabella presents the most anterior part of the forehead (Figure 2). The sellion is located at the midline of the base of the nasal root. It is the most posteriorly located landmark of the frontonasal contour (Figure 2) [31].

### 3.2. Nasal Side Wall Scrunch Wrinkles (Vertical Lines)

**Figure 5:**
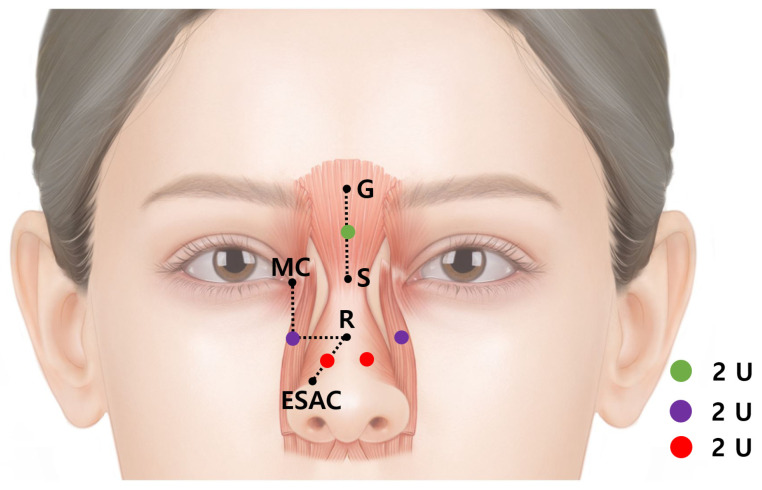
The injection point for the horizontal radix line (green dot) is in middle of the glabella (G) and sellion (S); for the nasal side scrunch wrinkles (red dots), it is in the middle of the rhinion (R) and the medial end of the supra-alar crease (ESAC); and for the bunny lines (purple dots) it is at the crossing point of the horizontal line at the level of the rhinion and the vertical line at the level of the medial canthus; 2 Units of botulinum neurotoxins should be injected per point.

Scrunch wrinkles on the nasal side wall are affected by the transverse part of the nasalis muscle. A dose of 2 U should be injected into the superior ala of the nose on both sides. The injection should be conducted in the middle of the rhinion and the medial end of the supra-alar crease (Figure 5).

### 3.3. Bunny Line (Oblique Nose Furrows)

The bunny lines are oblique wrinkles on both sides of the nose dorsum at a 45° angle. The lines are caused primarily by the levator labii superioris alaeque nasi muscle and secondarily by the medial muscular band of the orbicularis oculi muscle. A dose of 2 U should be injected into the upper part of the levator labii superioris alaeque nasi muscle on each side.

The injection should be conducted at the crossing point of the horizontal line at the level of the rhinion and the vertical line at the level of the medial canthus (Figure 5). 

## 4. Discussion

The nasal region has complex anatomical structures that may lead to adverse effects, such as BoNT rebalancing. The wrinkles in the nasal area can be exaggerated after BoNT injection because of this phenomenon. Therefore, it is important to identify and differentiate wrinkles in the nasal region. In addition, BoNT in the nasal region can cause major problems, such as diplopia resulting from the unintended blocking of the rectus inferior or medialis [4,5,32,33]. Although the incidence of BoNT causing diplopia is uncommon, it can be critical to some individuals [3,5]. Chen et al. reported a patient with BoNT in the lateral canthal region that caused lateral rectus paresis [4].

Side effects of BoNT injection in the upper nose region of the facial muscles, such as ptosis and samurai eyebrows, have been reported [31,32]. Although sensitivity to BoNT differs among individuals, there is no effective treatment for ptosis, which persists for several months [31,32]. Accurate BoNT injection points from an anatomical point of view have been suggested in various studies concerning BoNT injection in specific muscles [16,17,18,19,20,21,33,34,35,36,37]. According to the meta-analysis conducted by Camargo et al., most studies had duration of treatment of 5 months [38]. Notably, botulinum toxin effects take about two weeks to fully develop and last three to four months. 

Precise injection guidelines can directly relate to fewer BoNT injections. When increased doses and repeated BoNT injections are administered, antibodies can be produced, leading to inadequate treatment outcomes [6,7,8,9]. Therefore, an extensive and detailed anatomical understanding of the muscles is crucial to achieve maximum results with the lowest possible amount of BoNT. If the desired outcomes are not attained, an additional retouching treatment may follow. Likewise, during an injection procedure, manual blocking of the inner boundary of the orbital rim should be carried out [39]. The injection should be performed gently and slowly to prevent BoNT from laterally diffusing to the eyelids [40]. The limitation of this study is that the review is an anatomy-based proposal for nasal-region wrinkles. These precise injection methods would be time consuming to be applied in clinics. Moreover, the suggested doses are not universal to all types of BoNT and may applied in increased or decreased doses [41]. 

In summary, the suggested injection point for the horizontal radix line is at the middle of the glabella and sellion; at the middle of the rhinion and the medial end of the supra-alar crease for the nasal side crunch scrunch wrinkles; and at the crossing point of the horizontal line at the level of the rhinion and the vertical line at the level of the medial canthus for the bunny line. An amount of 2 U of botulinum neurotoxin should injected per point. 

This study carried out a broad analysis of published research on the anatomy of muscles in the nasal region to provide anatomical guidelines for BoNT indications. 

## Data Availability

Not applicable.
